# Fire Resistance Improvement of Fast-Growing Poplar Wood Based on Combined Modification Using Resin Impregnation and Compression

**DOI:** 10.3390/polym14173574

**Published:** 2022-08-30

**Authors:** Xiaowu Cheng, Dong Lu, Kong Yue, Weidong Lu, Zhongfeng Zhang

**Affiliations:** 1 College of Civil Engineering, Nanjing Tech University, Nanjing 211800, China; 2 College of Materials Science and Engineering, Central South University of Forestry and Technology, Changsha 410004, China

**Keywords:** fast-growing poplar, borate–phenol–formaldehyde resin, impregnation, compression, fire resistance

## Abstract

Fast-growing poplar with low wood density has been generally regarded as a low-grade wood species and cannot be used as a building material due to its poor fire resistance. As the fire resistance of wood materials is positively correlated with density, combined treatment using resin impregnation, which imparts thermal resistance, and compression, which improves density, appeared to be a route toward improved combustion performance. Fast-growing poplar wood was modified with a combination of borate-containing phenol–formaldehyde resin impregnation and compression in a transverse direction at varying intensities. The effects of the combined treatment on fire resistance were then examined and discussed. Char residue morphology analysis and microscopic observations were conducted to reveal the effects and mechanism of the combined treatment on fire resistance improvement. The test results showed that fire resistance was greatly improved, including the static and dynamic bending performance at elevated and high temperatures, as well as the combustion performance. The higher the compression ratio was, the better the fire resistance of the modified wood.

## 1. Introduction

In recent years, wood has been widely used in the construction industry and other industries for its superior aesthetic appearance, environment-adjusting characteristics, and high strength-to-weight ratio [1,2]. It should be noted that carbon sequestration in naturally renewable wood materials is prominent and carbon dioxide emissions extremely low compared to commonly used petrochemical building materials, such as steel, aluminum alloys, and plastic [3,4]. For decades, timber structures have greatly increased, which is attributable to the understanding of the effect of the environmental carrying load of the building industry on sustainability [5].

It should be pointed out that the growth cycle of natural trees is long, leading to an insufficient supply of high-quality structural wood materials. There is an imbalance between supply and demand for high-grade wood materials, which cannot meet the urgent demand for structural wood resources due to rapid economic and social development [6]. The emergence and use of fast-growing tree species has substantially overcome this disadvantage. The scientific and reasonable utilization of fast-growing wood in the building industry can effectively alleviate the tight supply of natural wood. Poplar is one of the most widely cultivated fast-growing trees because of its extremely short harvesting cycle. Particularly in China, it has been the most commonly used wood species [7]. Poplar wood is mainly cut to a small size for wood-based composites in the furniture industry in China, but it has been found that poplar wood has some undesirable inherent characteristics, including a relatively lower density and looser texture than natural woods [8], which are main factors governing mechanical strength [9]. Consequently, fast-growing wood species are not generally recommended for use in load-bearing building elements [2] and are thus severely restricted in structural applications on account of their strong hygroscopicity [10], weak decay resistance [11], creep resistance [8], poor dimensional stability [12], and especially their flammability and low strength class [1].

Therefore, it appears to be increasingly necessary to modify fast-growing wood, which would greatly expand its use in all aspects, especially in the construction industry with its huge demand for wood. Studies have been conducted to improve the undesirable aspects of fat-growing wood, and the most widely used modification has been resin impregnation and compression; the former generally includes a thermosetting resin with low molecular weight, such as urea–formaldehyde, phenol–formaldehyde (PF), or melamine–formaldehyde resin, and the latter increases wood density using compression in the transverse direction.

PF resin is widely used because of its good thermal resistance, durability, good flame retardancy, and low smoke quantity. In wood applications, PF resin has generally been employed as a good wood modifier for enhancing wood’s mechanical properties and fire resistance. It has been shown that borate has excellent oxidation resistance, and borate addition in the flame retardant effectively reduces flame spread. Therefore, borate was added in the synthesis of PF resin to further improve its antioxidant behavior at high temperatures [13]. The time to ignition and primary exothermic peak of Chinese fir impregnated with borate-containing PF (BPF) resin were 2.5 and 0.74 times greater compared to untreated specimens [9]. At the same time, the parallel-to-grain compressive and bending strengths of fast-growing Chinese fir with an air-dry density of 392 kg⋅m^−3^ were increased by 24.0 and 25.6%, respectively, after being impregnated with low-molecular-weight BPF resin. Further improvements in mechanical properties were found, and the two values increased by 117.0 and 107.3%, respectively, after being treated using PF resin impregnation and compression, compared to untreated wood [9]. The density, elastic modulus, and bending strength of PF-impregnated laminated veneer lumber made of Japanese cedar with a density of 0.32–0.36 g·cm^−3^ reaches 0.87 g·cm^−3^, 13 GPa, and 170 MPa, respectively [14]. Thermosetting resin penetrates easily into the cell walls and amorphous regions of cellulose fibrils [15,16]. At high-temperature, thermosetting resin within wood cures and forms strong chemical bonds between the resin and the wood. Most properties of wood impregnated with resin can be significantly improved, including its mechanical properties, as well as its decay and fire resistance, which have been mainly attributed to increased wood density [2,11].

The effects of compression are related to a number of factors, such as tree species, temperature, assembly method, and compression rate. Wang et al. [17] compressed poplar wood and found that the bending strength, elastic modulus, and hardness of the treated specimens were 1.69, 2.18, and 2.92 times that of the untreated samples, respectively. Jakob et al. [18] prepared partially delignified and densified spruce veneers. The flexural strength and elastic modulus of plywood was found to increase 2.4- and 3.5-fold, respectively, and poplar-plywood density increased from 550 to 840 kg⋅m^−3^ and was used as sheathing panels for light-frame shear walls in a previous study. The elastic lateral stiffness and ultimate bearing capacity of a shear wall sheathed with poplar plywood possessing higher marginal density increased by 47.8 and 37%, respectively [19]. It should be noted that wood density increases also contribute to improved fire resistance. For example, the ignition time of wood with a high density is longer and the charring rate lower compared to low-density wood [2], which is specified in fire-resistant designs for timber structures by Australian and European standards. 

The primary purpose of this study was to increase the fire resistance of low-value fast-growing poplar wood using a combined treatment of resin impregnation and compression. The mechanism of performance improvement was examined and discussed using microscopic observations.

## 2. Materials and Methods

### 2.1. Materials

Commercially available fast-growing poplar logs with relatively straight stems were obtained from the wood market in Jiangsu province, Eastern China. The trees were 11 years old and 383 mm in diameter at breast height. The logs were cut into dimensional lumber as knotless, noncracked, and normally grown lumber with a size of 38 × 140 × 3050 mm^3^ (radius, tangential, and length), and pieces were randomly chosen from sapwood with average annual ring widths between 28.5 and 31.5 mm. The average air-dry density of poplar wood was 0.444 g∙cm^−3^ at a 12% moisture content (MC). The average initial MC of the specimens was measured as 11–13% using an oven-drying method according to Chinese standard GB/T 1927.4 [20]. The specimens were conditioned at 20 °C and 65% relative humidity (RH) for more than two weeks, until MC equilibrium was achieved, before they were impregnated with resin or tested.

The specimens were randomly divided into three groups. The first group was the control and tested for static and dynamic bending strength at room temperature and fire behavior. The second contained specimens impregnated with resin, and the third group was modified with a combined treatment of resin impregnation and compression. The strengths and fire behavior of specimens in the second and third groups were assessed after modification. All the chosen test specimens had similar initial density and density differences within a group, controlled to no more than 5% after being conditioned to eliminate the effects on the selected properties.

PF resin was provided by a wood-based panel manufacturer (Landis Jiangsu Feiya Wood Co., Ltd., Xuzhou, China), which had a solid content of 51.5%. BPF resin was synthesized using chemical reactions of formaldehyde, phenol, and boric acid in the laboratory according to a previous study [1]. Formaldehyde (38.5%) was purchased from Xilong Chemical Co., Ltd. (Guangzhou, China). Sodium hydroxide (96%), boric acid (99.5%), and phenol (99%) were all obtained from Shanghai Shiyi Chemical Reagent Co., Ltd. (Shanghai, China). The produced BPF was light reddish brown in color and its original solid content and pH were 49.3% and 7.9, respectively. The resin was stored at 0–5 °C to slow its curing rate.

### 2.2. Wood Modification

Poplar boards with thicknesses of 20.0, 22.2. 25.0, 28.6, and 33.3 mm were prepared and pressed to a final thickness of 20 mm to obtain compression ratios of 0, 10, 20, 30, and 40% for bending strength tests at ambient temperature (20–25 °C) and 280 °C. Wood specimens with an original thickness of 12.5 and 16.7 mm were prepared for cone calorimeter tests of 10 mm-thick modified specimens, with a compression ratio of 20 and 40%, respectively. BPF resin for application was diluted to 30% (*w*/*w*) and homogenized for 5 min using a mixer running at a pumping capacity of 200 L·min^−1^ prior to each use. The specimens were then submerged in the solution in a high-pressure steel sealed capsule at 15 kPa vacuum for 4 h and further soaked at 0.45 MPa and 50 °C for 5 h. Finally, excess resin was removed under a 15 kPa vacuum for another half an hour. After treatment, the specimens’ MC was adjusted by drying the treated specimens in an oven at 60 °C for >10 h until the MC ranged from 15 to 20%. 

After that, the impregnated specimens to be compressed were heated and pressed using a universal hot-pressing machine (made in Qingdao, China) to allow for the polymerization of resin deposited within wood, as well as its compression. A temperature of 150 °C was employed according to the requirement of BPF resin curing [9]. The final thickness of the modified specimens was achieved using a gauge bar. The hot-pressing time was 2 min per mm. The treated specimens were then removed from the press and held to be conditioned in a closed environment at 20 °C and 65% RH until they achieved a constant mass. The wood specimens were cut into test pieces according to the requirements specified in the relative standards.

### 2.3. Characteristics Analysis of Resin

The analysis of BPF resin characteristics consisted of solid content, pH, and curing kinetics. The resin’s solid content was determined according to Chinese standard GB/T 14074 [21]. Five replicates were performed of each measurement and the average obtained. The determination of resin pH was conducted using a pH meter (PHS-3E, Shanghai INESA Scientific Instrument Co., Ltd., Shanghai, China) at room temperature. The curing characteristics of the resin were analyzed using a combined method of differential scanning calorimetric (DSC) measurement and kinetics. The resin was dried in a vacuum at 20 °C, ground to a powder, and assessed using DSC (200F3, Netzsch-Gerätebau GmbH, Selb, Germany). Dynamic scans were performed at heating rates of 10, 15, and 20 K·min^−1^ under nitrogen at 30.0 mL·min^−1^ and the scanning temperature was between 20 and 250 °C. The activation energy at the peak point of the DSC curves was obtained according to ASTM E689-18 [22], and calculated using the differential Kissinger method [23].

### 2.4. Bending Tests of Wood Specimens

Bending tests were conducted using static and dynamic loading methods. A previous study reported that the wood begins to char when the temperature reaches 280 °C [24], which was regarded here as the wood-charring temperature. In static bending-load tests, the bending strength of the control and modified specimens were tested at room temperature and 280 °C. The static bending specimens were cut to a size of 20 × 20 × 300 mm^3^ (width, thickness, and length), and bending strength (*σ_b_*) determined using Equation (1) according to Chinese standard GB/T 1927.9 [25], expressed as

(1) $$\sigma_{b}=\frac{3\cdot P_{\max}\cdot l}{2\cdot b\cdot h^{2}},$$

where *σ_b_* is the bending strength (MPa); *P_max_* is the ultimate bearing capacity (N); and *l*, *b*, and *h* are the span, width, and thickness of a test specimen (mm), respectively.

**Figure 1:**
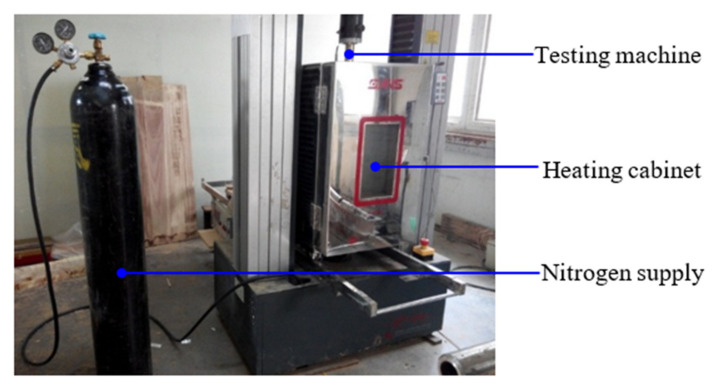
Test set-up for immediate bending strength at elevated and high temperatures.

The static bending tests were performed using an MTS electromechanical universal testing machine (SANS CMT5205, MTS Systems Corp., Eden Prairie, MN, USA) with an ultimate capacity of 300 kN (Figure 1). The loads were automatically measured and recorded by the universal testing machine, with a test considered completed when a specimen failed. The speed of loading was set at 3 mm·min^−1^, and 10 replicates were performed for each sample group.

Dynamic thermomechanical tests were carried out using a DMA (Q800, TA Instruments Ltd., New Castle, DE, USA) with tension, compression, shear, bending, and cantilever test capabilities. In this study, the dynamic thermomechanical behavior of the specimens was determined under a three-point bending-load and air atmosphere conditions. Specimen were cut to dimensions of 3 × 10 × 50 mm^3^ (thickness, width, and length) according to the internal dimension of the sample chamber and previous studies [26,27,28]. The temperature range was set from 20 to 310 °C and the speed of heating set at 5 °C·min^−1^. A sinusoidal alternating force with a maximum value of 0.6 N was loaded on the specimens with a vibration amplitude of 50 µm, and the results recorded automatically once per min. 

### 2.5. Fire Resistance Testing of Wood Specimen

In a fire, when the temperature reaches 280–300 °C, wood surfaces are converted to char, which has low conductivity and impedes further burning [24,29]. The charring layer retards heat transfer and thermal decomposition in the wood’s interior. Therefore, wood with a high temperature gradient within timber elements with a large cross-section, when loaded in the absence of oxygen, including the pyrolysis zone, determines the load-bearing capacity [30]. To reveal the mechanical properties of wood specimens at high temperatures, tests were carried out in an atmosphere box, which was flushed with nitrogen prior to being sealed, because oxidation accelerates wood decomposition reactions [31]. The mechanical properties of wood specimens exposed to 280 °C for 1 h were individually assessed while heated in an airtight atmosphere test box (GDX 300, MTS Systems Co., Ltd., Shanghai, China) with dimensions of 300 × 300 × 600 mm^3^ (width × depth × height, Figure 1). After the core temperature in the temperature specimens reached the target, the strength specimens were heated for another 1 h, followed by mechanical property testing in the heating cabinet. K-type thermocouples and a temperature patrol meter (DX1012, Yokogawa Test & Measurement Corp., Tokyo, Japan) were used to test the target temperature, with temperature acquisition once per min. The mechanical properties at high temperatures were conducted using an MTS electromechanical universal testing machine. The speed of loading was the same as in mechanical property testing at room temperature. Ten replicates were performed for each sample group.

Fire resistance testing of the control and modified specimens was conducted using Cone Calorimeter (FTT0007, FTT (UK) Co., Ltd., Derby, UK) according to ISO 5660-1 [32]. In a cone test, a 100 × 100 × 10 mm^3^ specimen (width × length × thickness) was placed in a sample holder in a horizontal position, with the back coated with a low-conductivity material to reduce heat loss to the sample holder. The distance between the sample and cone heater was set at 25 mm. The time to ignition (TTI), heat release rate (HRR), total heat release (THR), and mass retention (MR) were determined at an irradiance level of 50 kW·m^−2^ and data acquisition at once per second. Two specimens were tested in each group and averaged.

The limited oxygen index (LOI) was employed using Oxygen Index Tester (JF-3, Nanjing Jiangning Analysis Instrument Co., Ltd., China) in oxygen index tests, in which the specimen dimensions were 100 × 16.5 × 3 mm^3^ (length × width × thickness). In each parameter test, 15 replicates were performed under each condition and the mean values obtained.

The residual cross-section (char residue morphology) after a cone test was visually observed. The microscopic characteristics were examined using Scanning Electron Microscope (Phenom ProX, Thermo Fisher Scientific Inc., Pittsburgh, PA, USA). Two specimens were tested in each group and averaged.

## 3. Results and Discussion

### 3.1. Characteristics Analysis of Resin

**Figure 2:**
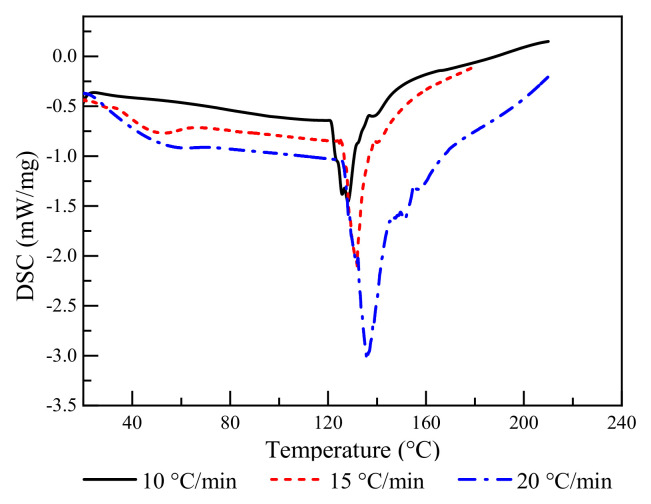
DSC curve of thermosetting BPF resin at different heating rates.

The solid content of BPF resin was 49.3% and the pH was 7.90, which were similar to and slightly lower than those of conventional phenol–formaldehyde resin [33]. The differential Kissinger method was based on thermal experiments with different heating rates to determine the kinetic parameters of a solid-state reaction. This non-isothermal method derived the activation energy ∆E using the peak temperature T_p_ at which the maximum reaction rate occurred. If the heating rate β was different, T_p_ changed correspondingly. The plot of ln(β/T_p_^2^) versus 1/T_p_ was a straight line (Figure 2), and the correlation coefficient *R*^2^ between the measured scatter plot and the fitted line was 0.936, such that linear fitting was shown to be applicable with relatively high accuracy.

The peak temperatures of BPF resin at heating rates of 10, 15, and 20 K·min^−1^ were 128.8, 133.5, and 137.4°C, respectively. The variation in T_p_ with β was only governed by ∆E, which was obtained by the relationship between ln(β/T_p_^2^) and 1/*T*_p_. According to the slope of the curve and calculations, the ∆E of the BPF resin was 55.79 kJ·mol^−1^ (Figure 2). The activation energy of PF resin used for wood impregnation has been reported to be between 58 and 105 kJ·mol^−1^ [23,33], indicating that the existence of borate was conducive to the self-condensation of PF resin. Thus, it was shown here to be beneficial to impregnate wood with BPF resin due to the reduction in energy consumption.

### 3.2. Bending Properties of Wood Specimens at Elevated and High Temperatures

#### 3.2.1. Static Bending Strength

**Figure 3:**
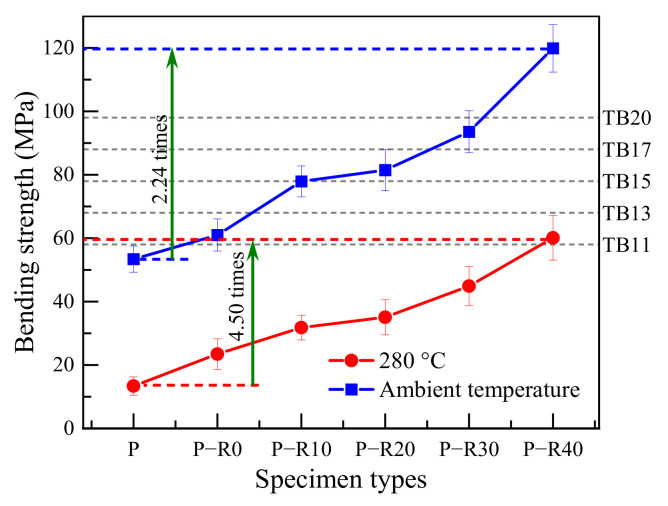
Bending strength of the control and modified poplar wood specimens at room temperature and 280 °C. P—control; P-R0, P-R10, P-R20, P-R30, and P-R40—BPF specimens with 0, 10, 20, 30, and 40% compression ratios, respectively.

Bending strength is a significant parameter for structural elements in construction. The cross-sectional dimensions of load-bearing materials are generally determined by strength class in structural design. The bending strengths of the control and BPF specimens with varying compression ratios were plotted and compared (Figure 3).

All the specimens under the room temperature condition were stress-graded and their strength classes determined by bending strength according to Chinese standard GB 50206 [34]; the detailed regulations are listed in Table 1.

The bending strength of the control was 53.40 MPa at room temperature and was the worst of all the specimen types (Table 1 and Figure 3). The control could not be used in structural building materials because its strength class did not meet the load-bearing demand according to Chinese standard GB 50206 [34]. Compared with the control, the bending strengths of the modified specimens with the combined treatment of resin impregnation and compression improved significantly. The bending strengths were increased by 14.23, 45.90, 52.58, 75.19, and 124.49% compared to the controls, after the resin-impregnated specimens were hot-pressed using compression ratios of 0, 10, 20, 30, and 40%, respectively. The BPF specimen strengths were positively related with the compression ratio, and their strength classes met the demand of strength classes of TB11, TB13, TB15, TB17, and TB20 according to Chinese standard GB 50206, respectively [34]. The results were very consistent with those in a previous study [9], and strength increases were attributed to the chemical bonding between the thermosetting resin and wood constituents.

From a molecular standpoint, cellulose is the most abundant wood chemical component and contributes significantly to wood strength. The low-molecular part of the resin was able to penetrate into the amorphous region in cellulose using the vacuum-pressure method and improved bonding between the cellulose microfibrils after curing. Meanwhile, the high-molecular part of the resin filled in the wood cell cavities and served as a mechanical support for the wood cells. Thermosetting resin bulked the fibers or, via chemical crosslinking, the cellulose chains of the component fibers, resulting in an effective improvement in bearing capacity and a significant reduction in deformation under load. Therefore, resin impregnation increased the wood strength. Furthermore, the mechanical properties of wood predominantly relate to wood density and were greatly improved after compression due to the increase in the volume proportion of the cell walls [14,15].

The impact of high temperature on immediate bending strength was clear, and reductions in bending strength at high temperatures in all types of specimen were observed (Figure 3). The average immediate bending strength of the controls was 13.37 MPa at 280 °C and represented 25.04% of the value at room temperature. The combined modification of resin impregnation and compression improved the bending strength at elevated and high temperatures and the higher the compression ratio was, the better the improvement in the bending strength at high temperatures. The residual immediate bending strengths of resin–compression-treated specimens with a duration of 1 h at 280 °C were 1.75, 2.38, 2.62, 3.36, and 4.50-fold those of the controls after the specimens were subjected to 0, 10, 20, 30, and 40% compression, respectively.

The published literature has shown that high temperature results in decreased mechanical properties of these wood materials [1,9,35,36]. Wood mainly consists of cellulose, hemicelluloses, and lignin, with cellulose mainly contributing to tensile strength and its thermal degradation occurring when the temperature is 200 °C and higher. Hemicelluloses and lignin both have a 21–25% proportion within wood, with hemicellulose playing the role of connecting cellulose and possessing lower heat resistance in the wood structure. The thermal degradation of lignin takes place at temperatures between 150 and 200 °C [37]. Therefore, the control had a significant reduction in mechanical properties at 280 °C because of the severe thermal degradation of the chemical constituents within the wood.

The fire resistance of poplar wood was shown here to be increased after being treated with BPF impregnation and compression, and the incremental improvement was positively related to the compression ratio. The increased density of the treated specimens and high bond energies of B-O built after BPF resin [26] led to the prevention of combustion. The residual immediate bending strengths of resin–compression-treated specimens at high temperature were improved greatly, and improvement increased with increased compression ratio.

#### 3.2.2. Dynamic Thermomechanical Analysis (DMA)

**Figure 4:**
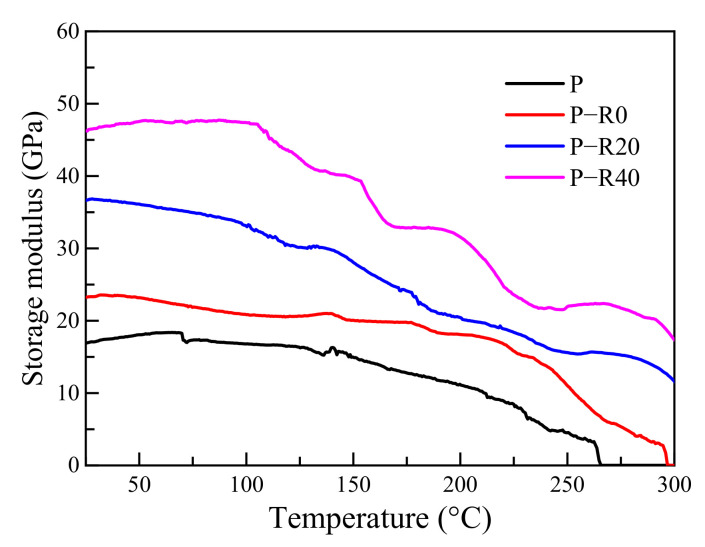
Storage modulus of control and modified wood specimens.

**Figure 5:**
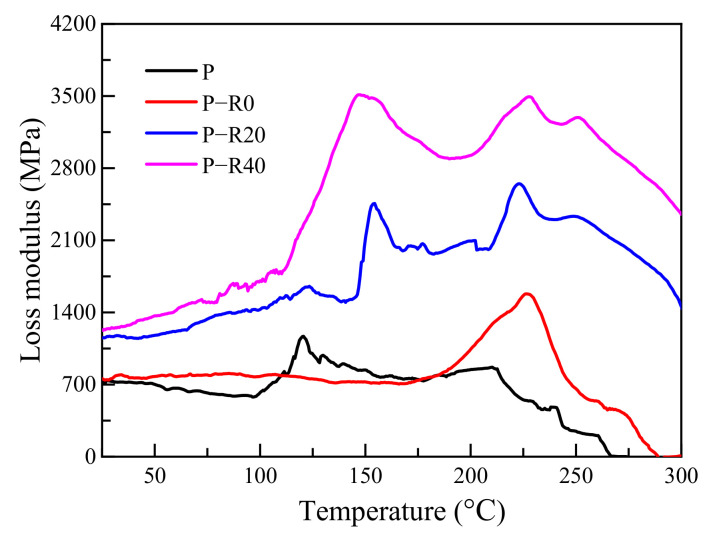
Loss modulus of control and modified wood specimens.

The storage modulus and loss modulus refer to elastic and viscous properties in the viscoelasticity of materials and are mainly used to characterize the stiffness and damping characteristics of a material, respectively. The greater the storage modulus was, the lower the deformation, indicating greater material stiffness. The loss modulus is positively related to material viscosity. The relationships between the storage and loss moduli and the elevated temperatures of the control and modified poplar wood specimens were compared (Figure 4 and Figure 5).

Differences among the poplar wood groups (the control and three modified groups) were apparent in several curves (Figure 4). The storage modulus of the control and modified wood specimens decreased by varying degrees with increased temperature, indicating that the materials tended to convert from a glass to a rubber state at elevated temperatures. The storage modulus curves of all the tested specimens with temperature had no relative maximum points. Under high-temperature conditions, the storage moduli of the BPF specimens with varying compression ratios were much higher compared to those of the controls.

Under relatively low-temperature conditions, the molecular motion energy in the main chemical compositions within poplar wood was insufficient, and only small molecules, such as molecular branches, main chains, or functional groups in the main chains and individual chain links stretched. This resulted in a relatively higher storage modulus [38], and deformation under a small load. The thermal movement of small molecules increased with temperature, and the chain segments began to move, resulting in reduced storage modulus. 

The elastic performance of the wood under a parallel-to-grain load was attributed to cellulose [27]. The lignin and hemicellulose primarily endowed wood plasticity [27,38], and were conducive to wood elasticity with a significantly lower contribution than cellulose [39]. In the case of the control, the initial storage modulus was 16.91 GPa at room temperature. The lignin content in poplar wood has been reported to be 23.8 wt% [40], which is much lower than that of conifer wood species commonly used in timber structures [41], such as Norway spruce at 30.9% [42], Scots pine at 28.8% [43], and fir (*Abies pectinata*) at 29.85% [28]. Fast-growing poplar wood cells have been found to consist of gelatinous fibers, which are not lignified. Therefore, the reduction in the storage modulus caused by lignin was not clear here. However, a rapid decrease in the storage modulus occurred at ~200 °C, caused by cellulose thermal decomposition. The initial storage moduli of BPF specimens with 0, 20, and 40% compression ratios were 1.37, 2.17, and 2.74 times those of the control, indicating that the combined modification of resin impregnation and compression contributed to increasing the storage modulus. BPF resin penetrated into the wood cell walls and chemically reacted with active functional groups, supporting the cellulose skeleton and improving stiffness. BPF resin contributed to producing higher mechanical properties at elevated and high temperatures due to good thermal resistance [1,9]. The reduction in the storage modulus of all the modified specimens decreased with temperature, which was similar to that of the controls. This was caused by thermal expansion of the cell walls, resulting in the reduction in intermolecular forces at medium temperatures [44], and the thermal decomposition of primary chemical constituents and weakening of support for the cell wall skeleton.

There were two clear sharp increases in the curve of loss modulus vs. temperature at ~120 and 210 °C, respectively, which indicated that the control had two viscosity transformation processes in the temperature range (Figure 5). Wood viscosity was mainly determined by lignin and hemicellulose [35,36], in which lignin generally showed more viscosity than cellulose and hemicellulose [45]. Several studies have reported that, under fully dry conditions, thermal softening occurs in lignin between 130 and 205 °C, in hemicellulose between 150 to 220 °C, and in cellulose between 200 and 250 °C [46,47]. The higher the MC, the lower the temperature at which the lignin softens. Salmén observed that lignin’s glass-transition temperature is 115 °C at 5% MC [48]. Here, under relatively low-temperature conditions, the loss modulus of the controls remained stable with temperature, indicating that viscosity was constantly accumulating. The free water within the wood vaporized at ~100 °C and lignin and hemicellulose thermally softened, resulting in an increased loss modulus, eventually reaching a peak value. The relative maximum point belonged to the glass-transition temperature of the controls. The MC and wood viscosity decreased with temperature, thus causing the loss modulus to decrease. Therefore, the first viscosity increase at 120 °C was mainly attributed to lignin’s thermal softening. The relatively slight increase in the loss modulus in the temperature range between 160 and 200 °C was caused by mild thermal degradation of hemicellulose [49]. Thermal decomposition of cellulose occurred at 200 °C and above [49], resulting in the second relative maximum point at ~210 °C.

In the case of wood specimens impregnated with BPF resin, the curve of loss modulus *vs*. temperature had one peak at ~226 °C and a peak at ~150 °C, which was not observed in the control. It is reported that low-molecular PF resin can penetrate into the amorphous areas of wood cell walls and can be cured by cross-linking with wood components, thus leading to retarded movement of the small molecular chains in the main chemical wood components. Two peaks at 155 and 225 °C were observed for modified specimens with resin impregnation and compression. Compression forced the wood cell walls together, resulting in an increased number of molecular chains per unit volume, thus decreasing the thermal motion space of the molecular chains and increasing the internal friction, as well as heat loss. The peak temperature of all the modified specimens increased significantly, indicating that the polymer chain length in the modified wood was lengthened. These findings agreed with reports by Ling et al. [50]. In the case of the specimens with compound modification, the first peak at the lower temperature represented links between short molecular chains, and the second peak at the higher temperature corresponded to links among long molecular chains.

### 3.3. Fire Performance

#### 3.3.1. Limiting Oxygen Index and Time to Ignition

**Figure 6:**
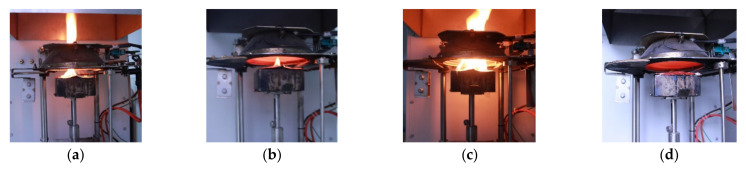
Burning phenomena in cone calorimeter tests. Initial ignition of all specimens during initial period (**a**), stable burning at relatively low speed (**b**), post-ignition violent combustion (**c**), and gradual burn to end (**d**).

In cone calorimeter tests, the visual burning behavior was similar among all specimens. The whole combustion process consisted of four stages: ignition, flame weakening, flame strengthening, and extinction. The burning behavior in the cone calorimeter tests is shown in Figure 6. The characteristic average values of the fire resistance testing are listed in Table 2.

All the tested specimens rapidly ignited during the initial period of burning, followed by relatively stable burning with a low speed. Violent combustion was observed again during the later period and the specimens gradually burned to an end. However, notably, the appearance time and intensity of the second ignition were clearly different between specimen types. The modified specimens exhibited a later post-ignition phase and relatively mild combustion compared to controls. The greater the degree of wood modification was, the more clear the improvement.

**Figure 7:**
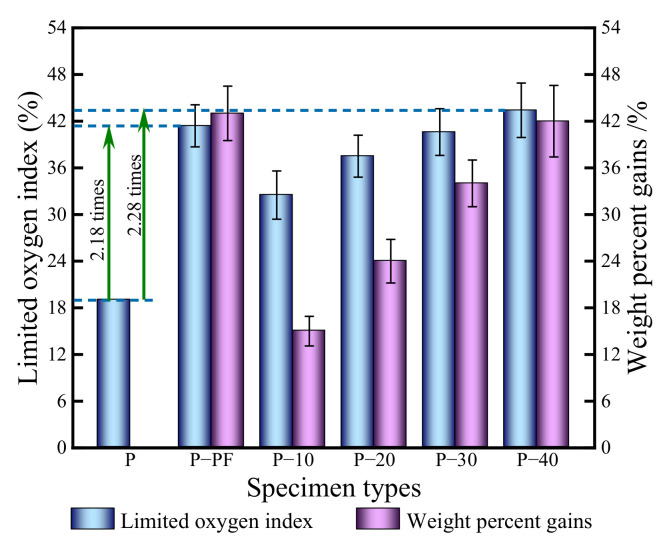
Limiting oxygen index of control and modified wood specimens. P—control; P-PF—specimens with 40% PF concentration; P-10, P-20, P-30, and P-40—BPF specimens with 10, 20, 30, and 40% resin concentration, respectively.

**Figure 8:**
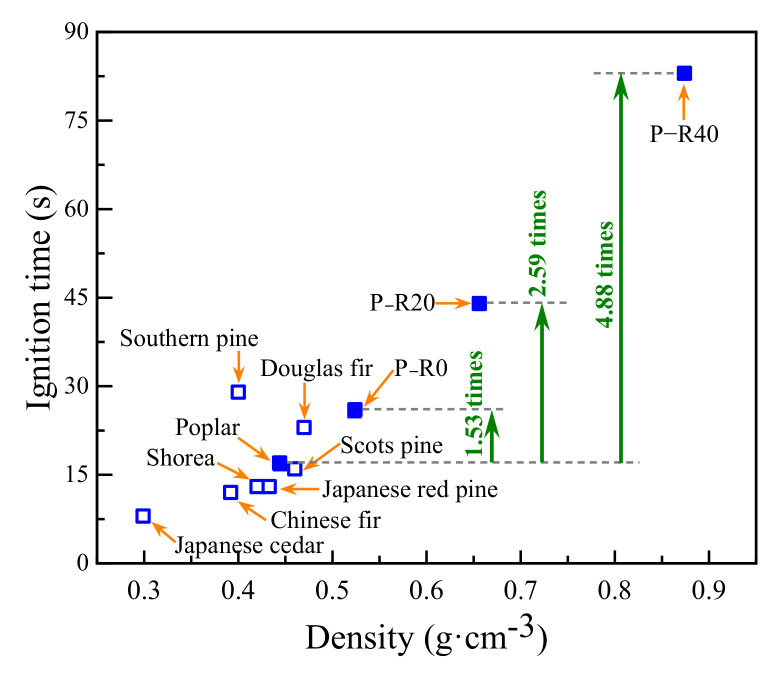
Ignition time of different wood species and modified poplar wood specimens.

The LOI represents the oxygen content when the material can just support its combustion in the mixture of oxygen and nitrogen. TTI refers to the time required for the specimen surface to be ignited in the set environment to stable combustion. The LOI and TTI are regarded as important indices for evaluating the combustion performance of building materials [51]. The greater the LOI, the less likely the material is to burn. The longer the TTI, the less flammable the material is, generally. In all cases here, the LOI and TTI were <50 s and 100 s, respectively. However, a clear difference in the LOI and TTI among all the wood specimen types was observed in the combustion tests. The LOI of the control and the specimens impregnated with resin under the absence of compression are shown in Figure 7. The TTIs of the wood specimens tested here were compared to those reported in the literature under the same heat flux condition (Figure 8).

The LOI of the control was the lowest and the LOI of the treated specimens increased with resin concentration (Figure 8). The LOIs of the BPF specimens increased significantly after resin impregnation and were 1.71, 1.97, 2.14, and 2.28 times those of the control after impregnation with a 10, 20, 30, and 40% resin concentration. The PF specimens had an LOI of 41.4%, representing a 2.18-fold increase compared to the controls. Under the same resin concentration, the percentage weight gain of the BPF specimen was 42.0% and slightly lower than that of the PF specimens (43.4%), but they had a relatively higher LOI. This difference might be attributed to the flame-retardant effects of borate within the BPF resin.

Japanese cedar with the lowest density (g·cm^−3^) was the most flammable due to its shortest TTI, while the other wood species were slightly nonflammable because of their relatively longer TTI (Figure 8). Xu et al. indicated that the densities of Douglas fir, Scots pine, Southern pine, and Shorea wood are 0.47, 0.46, 0.40, and 0.42 g·cm^−3^ and their TTIs were 23, 16, 29, and 13 s, respectively [52]. Japanese cedar, Chinese fir, and Japanese red pine have densities of 0.299, 0.392, and 0.433 g·cm^−3^ and their TTIs are 8, 12, and 13 s [9], respectively. The density of the controls was similar to that of Shorea and Scots pine, leading to a comparable TTI. The modified poplar specimens were the least flammable and exhibited longer TTI, especially in BPF specimens with a 40% compression ratio, exhibiting an LOI of 89 s, which was 5.24 times that of the control.

The experimental results confirmed that the combined treatment used in the present study effectively inhibited wood ignition.

#### 3.3.2. Heat Release and Fire Growth Index

The heat release evaluation included HRR and THR, which are two indices for evaluating the fire resistance of building materials. HRR refers to the amount of heat release from the specimen per unit area under a constant imposed heat flux, and the peak-HRR value is generally used to express fire intensity [51]. THR is the integral value of the released heat at every moment during the combustion of a specimen under set experimental conditions. The higher HRR and THR, the more heat generated, resulting in faster material pyrolysis and increased production of volatile combustibles, thus accelerating flame propagation. Therefore, these parameters are the most important and are generally used to determine the burning behavior of building materials. Two commonly used fire growth indices (FGI) have been adopted to evaluate the heat risk from building materials exposed to fire. One is the ratio of the value of the initial peak intensity of the HRR curve and TTI [53], and the other is the ratio of the value of the primary peak intensity of the HRR curve and its appearance time [54]. The lower the FGI, the more severe the burning will likely be and the worse a material’s fire resistance. 

**Figure 9:**
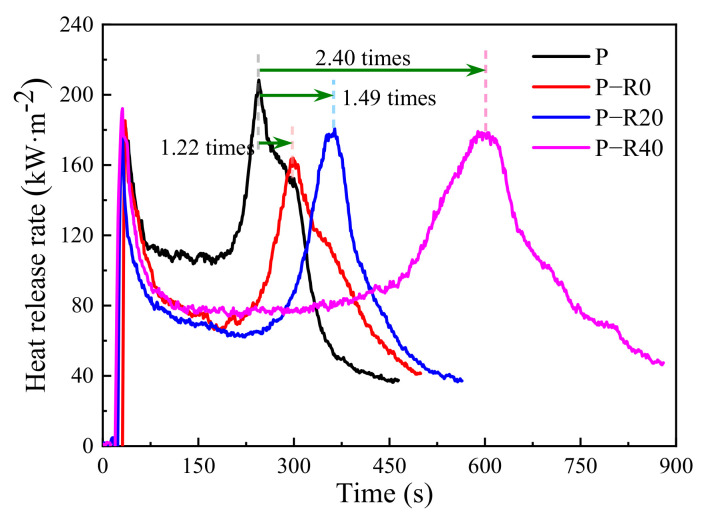
HRR of control and modified wood specimens.

**Figure 10:**
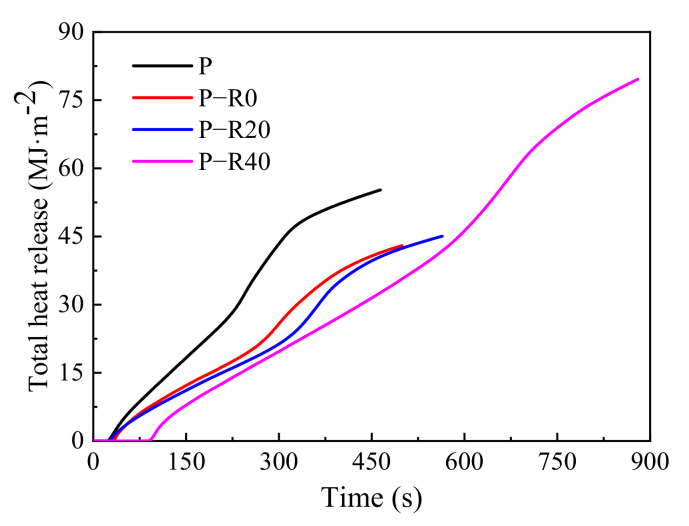
THR of control and modified wood specimens.

However, wood materials generally have two clear exothermic peak values with different intensities in the HRR curve. Here, the duration of the first exothermic peak was short, with the second, always regarded as the primary exothermic peak, having a longer duration [1,8,53]. The second exothermic peak with higher intensity is a serious threat, and essentially affects personnel escape and building fire safety. Therefore, the second exothermic peak was regarded as the primary peak intensity in this study. The HRR, THR, and FGI of the control and modified poplar wood specimens in the cone tests were compared (Figure 9 and Figure 10 and Table 2).

All HRR curves increased rapidly when wood specimens were ignited; then, they dropped and remained relatively constant for a period, and then, increased again (Figure 9). Two clear exothermic peaks over time were observed in the HRR curves of all the poplar wood specimens, which was similar to the published reports [47,48], and were defined as a first peak at the initial burning stage and the second peak before flame out [49,50]. The surfaces of the wood materials were first subjected to heat radiation and thermally decomposed into combustible gases at charring temperature and above. The continuous combustion reaction occurred with time and the first exothermic peak appeared, corresponding to the experimental behavior observed in Figure 6a. The first exothermic peak was relatively low since it led to the formation of a surface charring layer (Figure 6a,c). Therefore, after the occurrence of the first exothermic peak, HRR tended to become stable due to an exterior charring layer (Figure 6b), which had low conductivity and served as an effective barrier for reducing further violent burning in the interior burning area [21]. With continued burning, the HRR increase started because of heat accumulating at the back of the wood specimen at a high temperature [51]. The second exothermic peak is clearly evident in all the HRR curves (Figure 9). The wood specimens were not completely burned, and thus, the second exothermic peak was not instantaneously completed because of the simultaneous development of an exterior charring layer.

The first exothermic peak-appearance time of all the specimens ranged from 30 to 35 s, and the first exothermic peak value between 183.97 and 192.09 kW·m^−2^ (Figure 9 and Table 2). There was little difference between the control and the modified specimen groups. Although the wood was impregnated with resin and compressed, improvement in the first exothermic peak and its appearance time were not clear and all the specimens burned rapidly in the cone tests. During the stable combustion stage, the average HRR of all the modified wood specimens was ~75 kW·m^−2^, representing ~70% of that of the control (~105 kW·m^−2^) after the first exothermic peak. The second exothermic peak of the control was 208.38 kW·m^−2^. After being modified, the values of the specimens decreased to between 163 and 180 kW·m^−2^, which was a reduction of 13.3% and higher compared to the control. It should be noted that there was a great difference in the second exothermic peak’s appearance time among the tested specimens, with the second exothermic peak of the control occurring at 245 s. Modification with a combination of chemical and physical methods substantially created a greater and denser charring formation, which changed the materials’ pyrolytic chemistry and improved their thermal insulation, resulting in an improved protective effect of the exterior charring layer. The wood’s interior region had a relatively slow heating rate and released fewer pyrolytic products, causing a great delay in the second exothermic peak’s appearance time. The second exothermic peak’s appearance times for the BPF specimens were 1.22, 1.49, and 2.40 times those of the controls at 0, 20, and 40% compression ratios, respectively. In addition, according to the recommendation by Harada [55], the appearance time of the second exothermic peak is considered the fire endurance of wood-based materials. Great improvement was observed here in the fire resistance of poplar wood after using this combined treatment (Figure 9).

Control specimens with a thermal exposure of 464 s had a THR of 55.24 MJ·m^−2^ in the cone tests, which was higher than that of the untreated Chinese fir (38.88 MJ·m^−2^ after 503 s duration, Figure 10). The THRs of the BPF specimens with compression ratios ranging from 0 to 40% were all less than those of the controls. The greater the compression ratio was, the lower the THR. The THR of the BPF specimens with a 40% compression ratio approached 33.62 MJ·m^−2^ at ~464 s under constant heat irradiation, which was 0.59-fold compared to the control. Notably, the improvement increment of compound modification was better than that in a single treatment of resin impregnation. The conclusion was drawn that combined modification effectively suppressed the heat release of wood combustion, which would be beneficial for escaping and performing rescues in a building fire.

The FGI of the control was 0.09 according to the conventional equation [53], and the FGI of BPF specimens with 0, 20, and 40% compression ratios were 0.14, 0.24, and 0.43, representing 1.52, 2.58, and 4.68-fold increases, respectively, compared to the controls. Using the regulation related to the primary exothermic peak in this study [54], the FGI of the control was 1.18 and the FGI of the BPF specimens with 0, 20, and 40% compression ratios increased 1.82, 2.01 and 3.29-fold, respectively.

The fire resistance of modified poplar wood specimens was greatly enhanced compared to the control. Resin impregnation and compression contributed to a reduction in the primary exothermic peak and THR, and also delayed the peak-appearance time. The FGI of the modified specimens increased greatly no matter which calculation method was adopted, suggesting that the combined modification could reduce fire risk and the present treated wood exhibited improved fire resistance.

#### 3.3.3. Carbon Dioxide and Total Smoke Production

**Figure 11:**
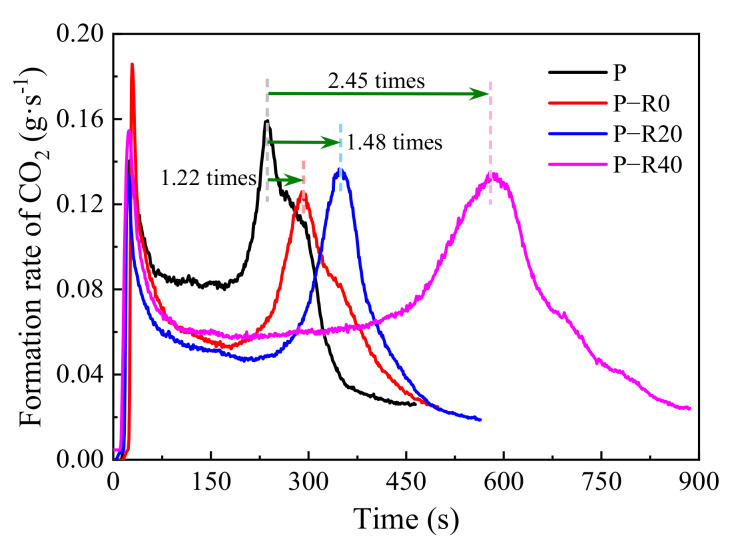
Carbon dioxide production of control and modified wood specimens.

**Figure 12:**
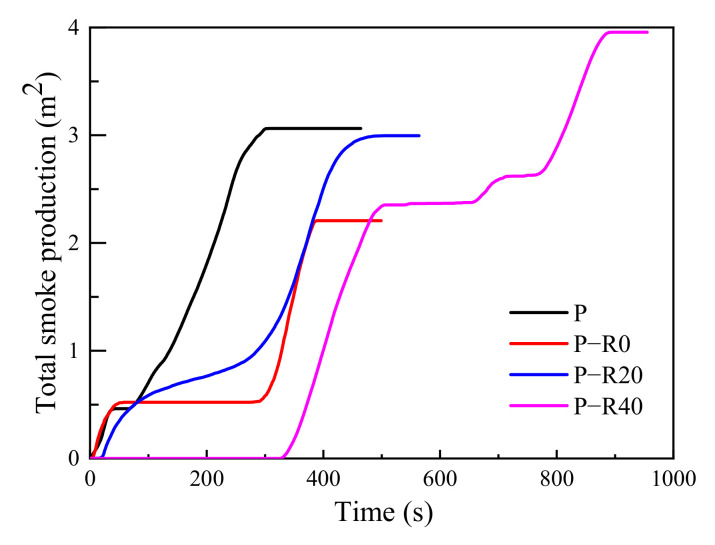
TSP of control and modified wood specimens.

The primary chemical constituents within wood materials are all flammable, which can lead to the formation of a charring layer and the release of volatile intermediates (carbon dioxide, CO_2_, carbon monoxide, methanol, acetic acid, and other toxic gases) [56]. Dense CO_2_ and smoke cause oxygen deficiency and disorientation in humans and are respiratory irritants generally regarded as a major factor in fire casualties [53]. Here, CO_2_ and the total smoke production (TSP) of untreated and modified wood specimens were recorded in the cone tests (Figure 11 and Figure 12 and Table 2).

The curves of CO_2_ formation rate over time had a similar shape to those of the HRR curves (Figure 9 and Figure 11), indicating that the heat release of the control and modified wood specimens subjected to high temperatures could be mainly attributed to CO_2_ formation. The CO_2_ formation curves of all the tested specimens had two clear peaks, representing the thermal degradation of chemical constituents at high temperatures, with the second one primarily due to high proportions of combustibles. The first CO_2_ formation peak of all the tested specimens was clearly observed in the cone tests. Afterward, CO_2_ formation clearly decreased and was stable at a loading of 0.081–0.085 g·s^−1^ (the control). The CO_2_ formation of the modified specimens at the stable stage ranged from 0.05 to 0.06 g·s^−1^. With increased time, the second peak occurred. The difference in the first peaks between the controls and modified specimens was not clear. However, the second peaks of the modified wood specimens appeared later compared to controls. The reductions in the second peak values of the modified specimens were 15–21% less than those of the controls, and the appearance times were 1.22, 1.48, and 2.45 times those of the controls at compression ratios of 0, 20, and 40%, respectively. Compared to the controls, with a TSP of 3.06 m^2^ at a loading of 464 s heat duration at a high temperature in the cone tests, the modified specimens produced significantly reduced total smoke formation, especially at a 40% compression ratio.

#### 3.3.4. Mass Retention (MR)

**Figure 13:**
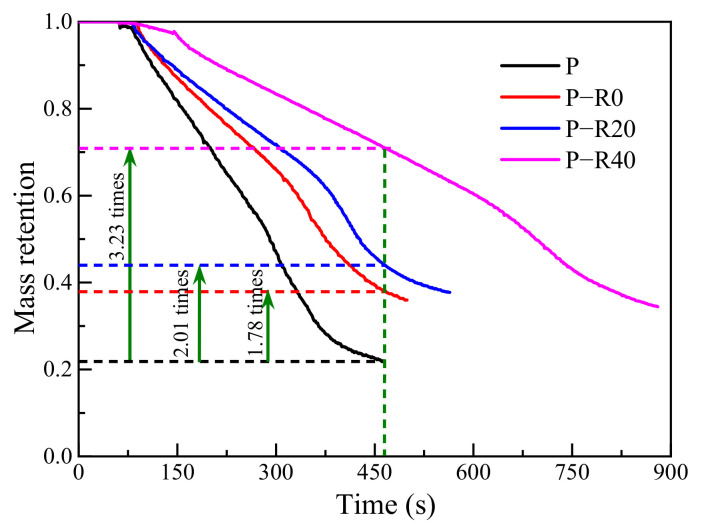
Mass retention rates of control and modified wood specimens.

MR is a ratio of the immediate residue mass during the burning process to the initial mass, which was automatically weighed and calculated in the cone calorimeter tests. MR reflects the degree of wood pyrolysis, volatilization, and burning, when being exposed to a constant heat flux. MR is highly correlated with heat release and total smoke release. A higher MR is indicative of a lower propensity for flame spread and can be used as an important index for the assessment of post-fire behavior. Generally, the greater the MR, the higher the residual bearing capacity after a fire. The evolution of immediate mass with time for the control and modified wood specimen groups is shown in Figure 13. 

The MR of all the tested specimens gradually decreased with time, indicating increased thermal decomposition of substantial wood constituents. The reduction in mass of the modified wood specimens was significantly lower than that of the controls. The MR of the control was 22.02% under a 464 s thermal exposure condition. In comparison, the initial values of the BPF specimens with 0, 20, and 40% compression ratios were 38.20, 44.21, and 71.09%, when subjected to the same thermal radiation duration, representing 1.78, 2.01, and 3.23-fold increases, respectively, compared to the controls. The difference in MRs between the controls and the modified specimens was clear, showing that the combined treatment used here greatly contributed to improving the fire resistance of poplar wood specimens. The MR of the modified specimens continuously increased with increased compression ratios of the BPF specimen. In the case of BPF specimens with a 40% compression ratio, the MR was 34.42% at a level of 880 s thermal radiation, which demonstrated that the combined treatment of resin impregnation and compression was conducive to improving wood fire resistance. The greater residues of the modified wood specimens were due to the superior thermal resistance of BPF resin and the dense structure endowed by compression, which thus reduced the wood pyrolytic rate. The positive effects on MR of the combined treatment were consistent with the heat- and smoke-release results.

#### 3.3.5. Char Residue Morphology and Microstructure Analysis

**Figure 14:**
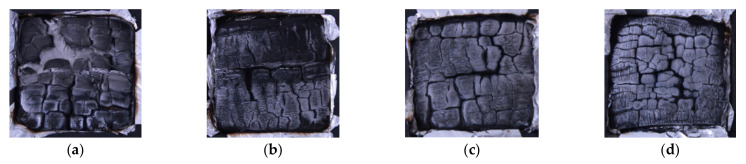
Residual cross-sections of a control (**a**) and BPF specimens with 0, 20, and 40% compression ratio (**b**–**d**, respectively) after cone testing.

The mechanical properties of these wood specimens seriously decreased with increased temperature, with specimens heated at 280–300 °C turning into a charring layer, which is generally considered to have no strength [57]. However, char has superior insulating performance due to its low conductivity and prevention of further burning from the unburnt interior [57]. Therefore, a charring layer is important in providing sufficient fire resistance in timber structures. The char residue morphologies of the control and modified wood specimens are shown and compared in Figure 14 after cone testing.

All these wood specimens produced a charring layer, and cracking with a relatively regular appearance occurred in all the specimens in cone testing (Figure 14). However, the charring residue morphology was greatly different among the groups of wood specimens. 

The controls appeared much looser and darker than other specimen groups, with the cracking being wider and deeper (Figure 14a). Some through cracking occurred, leading to a loss in the integrity of the charring layer, which separated into several pieces. The control was thus rendered into a small amount of grey ash residue, indicating relatively poor fire resistance. With an increased compression ratio, the charred residues of BPF-densified specimens remained intact, appearing denser and lighter due to fewer and thinner cracks (Figure 14b–d). This was observed because of resin deposited in the wood cell cavities and compression, which both effectively increased the charring layer density, indicating increased oxygen exclusion and decreased thermal conductivity and combustibility. Additionally, no ash was found on the residues of any of the modified specimens. The charring extent of the modified specimens was slight compared to controls. Thus, these major char observations from cone testing, as well as TTI, HRR, THR, and TSP, indicated a clear improvement in fire resistance when resin impregnation and compression were used together (Figure 4, Figure 5, Figure 6, Figure 7 and Figure 8).

BPF resin provided thermal resistance for this wood at elevated and high temperatures [58]. The modified specimens only slightly charred compared to the control, which was attributed to the positive effects of thermosetting resin filling the wood cell lumen and cell walls. Especially after being compressed, the density of the BPF specimens was greatly increased; this resulted in a dense charring layer at charring temperatures, which would retard the heat transfer and thermal decomposition of the wood interior. A char layer would impede the generated combustible gases from easily escaping from the interior, and the internal wood would thus retain relatively high strength. Thus, greater MR could provide a greater barrier under fire conditions. Therefore, the fire resistance of treated poplar wood with the combined modification of resin impregnation and compression was significantly improved, especially under high-compression-ratio conditions, suggesting better fire safety.

**Figure 15:**
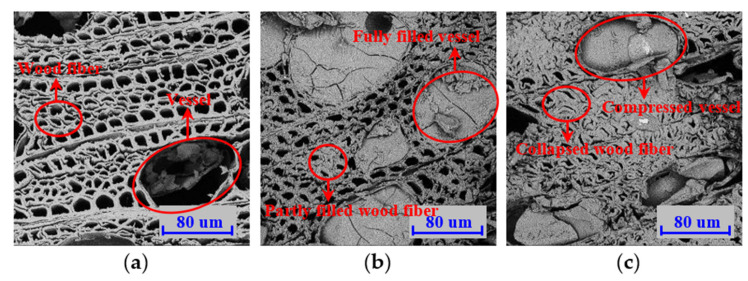
Transverse SEM images of control (**a**) and BPF specimens with 0 (**b**) and 20% (**c**) compression ratios prior to cone tests.

**Figure 16:**
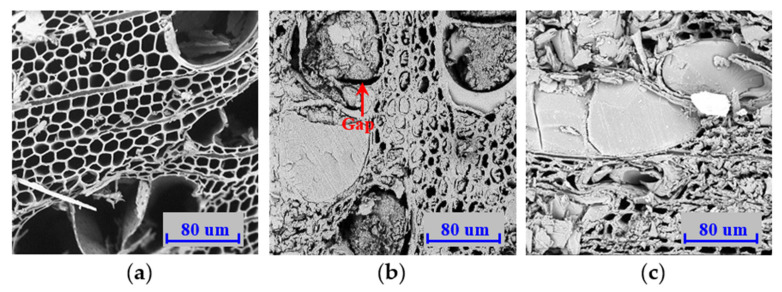
Transverse SEM images of control (**a**) and BPF specimens with 0 (**b**) and 20% (**c**) compression ratios after cone tests.

Microscopic observation of the modified wood before and after being heated is an essential indicator for revealing the thermal effects of BPF resin impregnation and of the mechanisms of cell deformation due to densification on mechanical properties and fire resistance at the cellular level. Therefore, microstructural investigations and analyses were conducted to provide insight into the effect of the combined modification on selected properties of the treated wood after the modification of different processing conditions. Electron microscopy was focused on wood specimen cross-sections, where most of the anatomical elements are shown in transverse directions. The microscopic images of the control and impregnated wood specimens prior to and after cone testing are presented in Figure 15 and Figure 16, respectively.

SEM analysis showed the microstructure of the controls (Figure 15a) and BPF specimens with 0 and 20% (Figure 15b,c) compression at a magnification of 1000×. Different from the orderly arrangement of rectangular tracheids in coniferous wood, the fiber-tracheid pattern in poplar wood is less regular and manifests as a pentagonal or nearly circular pattern. The large vessels here were visible and clear, evenly distributed in the growth ring, and possessed the morphological features of diffuse-porous wood species. The fiber-tracheid cells had a greater cell wall thickness and a smaller cell volume compared to those of the vessel cells. The vessel cells were thin-walled and oval-shaped in the transverse section and the pore area dozens of times greater that of the fiber-tracheids. The volume ratio of the fiber-tracheid-to-vessel cells was relatively low, leading to the poor strength of poplar wood.

The experimental setup and resulting images clearly exhibited that it was possible to observe resin deposition in wood tissue. Resin penetrated the wood to varying extents and the morphology of wood cells changed after being impregnated and compressed. This was clearly visible using SEM due to the feature of optical sectioning (Figure 15 and Figure 16). Microscopic images indicated that fiber-tracheids were partially or completely filled with resin (Figure 15b), and almost all the vessels fully filled with resin, suggesting that a chemical relationship between the constituents within wood and thermosetting resin had been built. Because there was so much resin deposition in the wood cells, the density increased and desirable performance improved.

The wood cells deformed along the direction of compression, and the cell lumen volume significantly decreased, which resulted in cell wall collapse. To obtain compressive deformation in wood material without a reduction in strength through clear damage to the cell, matrix constituents (lignin and hemicelluloses) should be in a rubbery state, above the glass-transition temperature [53,59]. According to the literature [13], the compression temperature should be 150 °C and the MC should be at least 5% to reach the glass-transition temperature of the matrix constituents. Thus, a compression temperature of 150 °C and an MC ranging from 15 to 20% were used in the present study and no clear cracking in the deformed wood cells was observed in any specimen cross-sections due to the sufficient plasticization. The plasticizing effects that the phenol-based resin, the relatively high temperature, and the moisture within the wood all had on the cell walls appeared to effectively reduce internal stresses during densification [54]. The cell lumens of the fiber-tracheids nearly disappeared and there was a clear reduction in vessel size along the compression direction (Figure 15c). Therefore, the selected properties of low-grade poplar wood were improved by the combined treatment of resin impregnation and compression. The BPF specimens at a 20% compression ratio had much smaller cell lumens compared to those of the BPF specimens without compression (Figure 15b,c), thus confirming further increased density in the compressed specimens compared to the controls and those with no compression. 

From microscopic analysis, it was evident that cells after compression were deformed without clear cracking and dislocations of the cell walls, and there was sufficient deposition of BPF resin in the cavities of the wood cells. This confirmed that the present method (resin impregnation and compression) was effective for the improvement of selected properties in low-value fast-growing wood. From microscopic images, wood cell thickness after the combustion performance tests grew thinner; this was caused by thermal decomposition of the chemical compositions and cellulose microfibrils, which were close together due to water loss at high temperatures, and was consistent with the findings in several studies. A significant decreased infrared band at 3406 cm^−1^ has been observed at high temperatures, suggesting the thermal degradation of hydroxyl groups in the hemicellulose of heated wood [60]. Yue et al. [61] reported that the cell wall thickness in Douglas fir latewood decreased from 8.0–9.1 µm at room temperature to 4.0–4.8 µm at 280 °C, mostly caused by thermal degradation of the wood composition. The hemicellulose content of *Populus cathayana* was determined to be 29.30% at room temperature and decreased to 9.17% at 180–220 °C [60].

Compared to the controls, there were no visually significant differences in the wood cells in the modified specimens before and after being heated. The cell structure of the heated wood remained intact (Figure 16a), which was attributed to the supporting effect of the superior thermal resistance of BPF resin in the cell lumens and walls. However, it should be noted that cured thermosetting resin in the cell lumens was separated from the cell walls in the heated specimens (Figure 16b,c). The wood chemical compositions were thermally degraded and the emission of water from the wood occurred at high temperatures, and wood cells then tended to shrink. This deformation was restrained by adjacent wood cells, resulting in the increased size of the cell lumens. 

It had been shown that borate forms impermeable glass coatings after thermal degradation, which excludes oxygen and prevents the further propagation of combustion [62]. Additionally, BPF resin had strong thermal resistance, which was significantly superior to the wood [58]. A clear gap between the resin and the vessels was generated, as well as with fiber-tracheids. Therefore, thermal-resistant resin impregnation and compression were concluded to be effective and contributed to the great improvement in wood fire resistance. Accordingly, the strength class of these modified specimens was greatly increased, the degree of pyrolysis was slight, and the time of pyrolysis significantly lagged compared to the controls.

## 4. Conclusions

The activation energy of BPF resin was found to be 55.79 kJ·mol^−1^ using the differential Kissinger method, and it was thus beneficial for impregnating wood due to its relatively lower energy consumption compared to conventional PF resin.

The combination of BPF resin impregnation and compression was attributed to increasing static bending strength and dynamic bending properties at elevated and high temperatures. The bending strengths of the BPF specimens at 40% compression were 2.24 and 4.50 times those of the controls at room temperature and 280 °C, respectively.

BPF resin impregnation and compression improved the fire resistance of poplar wood. The LOI, at 40% resin concentration, was increased 2.28-fold, and the TTI 4.88-fold. In the case of the modified specimens at compression ratios of 40%, the appearance times of the primary HRR, CO_2_ formation rate, and MR at a 453 s duration were 2.40, 2.45, and 3.23 times those of the controls, respectively. 

Microscopic observations showed that BPF resin deposited in the wood cell lumens and wood cells after compression were deformed without clear cracking, and that there were dislocations of the cell walls. The improved properties resulting from this combined treatment were attributed to the resulting structural integrity of the char residue.

The combination of resin impregnation and compression significantly improved the fire resistance of fast-growing poplar wood. The higher the wood density was, the better the improvement. BPF-treated poplar wood at a 40% compression ratio is thus recommended to be used as a load-bearing element in building construction.

## Figures and Tables

**Table 1:** Strength classes of structural hardwood according to Chinese standard GB 50206.

Wood Species	Strength Classes	Bending Strength (MPa)
Hardwood	TB11	58
	TB13	68
	TB15	78
	TB17	88
	TB20	98

**Table 2:** Fire resistance of control and modified poplar wood specimens.

**Specimen** **Type**	**Density** **(g·cm^−3^)**	**TTI** **(s)**	**HRR ^1^** **(kW·m^−2^)**	**THR ^2^ (MJ·m^−2^)**			**TSP (m^2^)**			**MR (%)**				**FGI**	
				464 s	499 s	564 s	464 s	499 s	564 s	464 s	499 s	564 s	880 s	[53]	[54]
P	0.444	17	208.38 (245)	55.24	-	-	3.06	-	-	22.02	-	-	-	0.09	1.18
P-R0	0.524	26	163.92 (299)	41.44	42.99	-	2.21	2.21	-	38.20	35.97	-	-	0.14	1.82
P-R20	0.656	44	180.66 (364)	40.55	42.39	45.06	2.96	2.99	3.00	44.21	40.84	37.74	-	0.24	2.01
P-R40	0.874	83	179.07 (589)	32.62	35.65	41.82	2.00	2.34	2.37	71.09	68.46	63.26	34.42	0.43	3.29

^1^ Data in parentheses—time to peak heat release rate (s). ^2^ Data in THR—second exothermic peak.

## Data Availability

Not applicable.
